# Supplementary material for: Coupling between the DEAD-box RNA helicases Ded1p and eIF4A
Source: eLife. 2016 Aug 5;5:e16408. doi: 10.7554/eLife.16408 (PMC4990422; doi:10.7554/eLife.16408)
Supplement: Supplementary file 1. — DOI: http://dx.doi.org/10.7554/eLife.16408.022 [file elife-16408-supp1.docx]

**Supplementary file 1**

**Materials**

1A – RNA Oligonucleotides

1B – Primers

1C – Plasmids

1D – Yeast strains

**Supplementary file 1A. RNA Oligonucleotides**

| **Substrate Name** | **Sequences** |
| --- | --- |
| 19 bp, 25 nt 3'-overhang | 5'- GCU GCG UCU UUA CGG UGC UUA AAA CAA AAC AAA ACA AAA CAA AA -3' ^(a)^ |
|  | 5'- A GCA CCG UAA AGA CGC AGC -3' |
| 16 bp, 25 nt 5'-overhang | 5'- AAA ACA AAA CAA AAC AAA ACA AAA U GC GUC UUU ACG GUG CU -3' |
|  | 5'- A GCA CCG UAA AGA CGC -3' |
| 16 bp, blunt | 5'- A GCA CCG UAA AGA CGC -3' |
|  | 5'- GCG UCU UUA CGG UGC U -3' |
| 16 bp, 10 nt 3'-overhang | 5'- GCG UCU UUA CGG UGC UUA AAA CAA AA -3' |
|  | 5'- A GCA CCG UAA AGA CGC -3' |
| 16 bp, 15 nt 3'-overhang | 5'- GCG UCU UUA CGG UGC UUA AAA CAA AAC AAA A -3' |
|  | 5'- A GCA CCG UAA AGA CGC -3' |
| 16 bp, 25 nt 3'-overhang | 5'- GCG UCU UUA CGG UGC UUA AAA CAA AAC AAA ACA AAA CAA AA -3' |
|  | 5'- A GCA CCG UAA AGA CGC -3' |
| 13 bp, 25 nt 5'-overhang | 5'- AAA ACA AAA CAA AAC AAA ACA AAA UAG CAC CGU AAA GA -3' |
|  | 5'- UCU UUA CGG UGC U -3' |
| 13 bp, blunt | 5'- UCU UUA CGG UGC U -3' |
|  | 5'- A GCA CCG UAA AGA -3' |
| 13 bp, 25 nt 3'-overhang | 5'- GCU UUA CGG UGC UUA AAA CAA AAC AAA ACA AAA CAA AA -3' |
|  | 5'- A GCA CCG UAA AGC -3' |

^(a)^ Underlined nucleotides indicate the duplex region.

**Supplementary file 2B. Primers**

| **Name** | **Purpose** | **Sequence** |
| --- | --- | --- |
| oAKH524 | *TIF1* overexpression vector | 5’ caaaagctggagctccaccgcggtggcggccgctctagaac TGGCCAGGAGAAACGAAGACGAGTGC 3’ ^(a)^ |
| oAKH525 | *TIF1* overexpression vector | 5’ cgacggtatcgataagcttgatatcgaattcctgcagcccg TGGCCAGATGTTGTCCCTGTTTATGCC 3’ ^(a)^ |
| oAKH526 | *TIF2* overexpression vector | 5’ caaaagctggagctccaccgcggtggcggccgctctagaac TGGCCATGACGACCACTATTACCAGC 3’ ^(a)^ |
| oAKH527 | *TIF2* overexpression vector | 5’ cgacggtatcgataagcttgatatcgaattcctgcagcccg TGGCCAATAGCGAGCCTCCTTTTGC 3’ ^(a)^ |
| oAKH575 | *DED1* overexpression vector | 5’ caaaagctggagctccaccgcggtggcggccgctctagaac TGGCCAGGCTAGCAGAATTACCCTCCACG 3’ ^(a)^ |
| oAKH576 | *DED1* overexpression vector | 5’ cgacggtatcgataagcttgatatcgaattcctgcagcccg TGGCCAAGGGAGAGAAAATGTAGGAAGGA 3’ ^(a)^ |
| X7 | Amplification of genomic *HIS3*-*DED1* region | 5' ATCGTTGAGTGCATTGGTGA 3' |
| X8 | Amplification of genomic *HIS3*-*DED1* region | 5' GTCACCATGAATGGCGGTAG 3' |
| X11 | Site-directed mutagenesis *ded1-95* allele | 5' GTCGAAATTAAGAGAATGGCAG 3' |
| X12 | Site-directed mutagenesis *ded1-95* allele | 5' CTCTTAATTTCGACAAAGATCAAAG 3' |
| X53 | Verification of *ded1-95* allele | 5' GCTACTTTTCCCGCTGATA3' |
| X54 | Verification of *ded1-95* allele | 5' GTCACCATGAATGGCGGTAG3' |
| Ded116delFt | *DED1*^117-604^ vector | 5’AAAGCATTTCATATTATGTCTTCTGGTATTAAC TTCGATAACTACGATGA 3' |
| Ded116delRs | *DED1*^117-604^ vector | 5’ TCTTGTTGTTCTTACGGAATACCACTTGCCAC 3’ |
| Ded116delFs | *DED1*^117-604^ vector | 5’TCTTCTGGTATTAACTTCGATAACTACGAT GATATTCCAGTGGACGC 3’ |
| Ded116delRt | *DED1*^117-604^ vector | 5’CATAATATGAAATGCTTTTCTTGTTGTTCT TACGGAATACCACTTGCCAC 3’ |
| zfDD10 | Amplification of wild-type *DED1* coding sequence form the yeast genomic DNA to insert into pBlueScript II KS(+) | 5'CCGTAGTGAGAGTGCGTTCAA3’ |
| zfDD11 |  | 5'CACATATTTGCTAGGCAACCC3’ |
| zfDD12 | Addition of tandem triple HA tags to the 3’-end of the *DED1* coding sequence in pBlueScript II KS(+) | 5’PO4GCCCGCATAGTCAGGAACATCGTATGGG TAGCCCGCATAGTCAGGAACATCGTATGGGTAC CACCAAGAAGAGTTGTTTG3’ |
| zfDD13 |  | 5’PO4TGATTTCAGACAAACTAGGG3’ |
| ugDD10 | Introduction of T408I mutation into *DED1* coding sequence in pET-22b(+)-*DED1* | 5‘ GTCGAAATTAAGAGAATGGCAG 3’ |
| ugDD11 |  | 5‘ CTCTTAATTTCGACAAAGATCAAAG3’ |
| zfDD14 | Amplification of *DED1*^117-604^ coding sequence from pET-22b(+)-*DED1* | 5’GGCT CTCGAG TCA CCACCAA GAAGAGTTGT TTGAAC 3’ |
| zfDD15 |  | 5’ CCAC GAG CTC TCTTCTGGTATTAACTTCGA TAACT 3’ |
| zfDD16 | Introduction of E171A mutation in eIF4A inpET-28a(+)-eIF4A | forward tailed primer 5'-CATCTTAGATGCAGCTGATGAAATGTTGTCTTCTGGTTTC3' |
| zfDD17 |  | forward short primer 5'AAATGTTGTCTTCTGGTTTCAAGGAACAAATCTA3' |
| zfDD18 |  | reverse tailed primer 5'ATCAGCTGCATCTAAGATGAACATCTTGATCTTGTCAGT3' |
| zfDD19 |  | reverse short primer 5'AACATCTTGATCTTGTCAGTTCTGAATCTA3' |
| zfDD20 | Amplification of eIF4A coding sequence from pET-28a(+)-eIF4A, to insert into pET-41a(+)and produce GST-eIF4A | 5’ CGGC CC GCG G GT ATGTCTGAAG GTATTACTGA 3’ |
| zfDD21 |  | 5’ GGTG CTCGAG TTAGTTCAAC AAAGTAGCGA3’ |

^(a)^ Underlined sequence matches pGP564 vector. Uppercase sequence binds the ORF to be amplified.

**Supplementary file 1C. Plasmids**

| **Name** | **Vector** | **Insert** | **Source** |
| --- | --- | --- | --- |
| pRP1555 | pRS413 (*HIS3; CEN*) | endogenous promoter *; DED1* | Hilliker et al., 2011 |
| pRP2048 | pRS413 (*HIS3; CEN*) | endogenous promoter; *ded1-tam* (deletion of amino acids 21-27,119-122, 531-540) | Hilliker et al., 2011 |
| pGP564 | pGP564 (*LEU2; 2μ)* | none | GE Healthcare |
| pAKH756 | pGP564 (*LEU2; 2μ)* | +/- 500 nucleotides around *TIF2* | This study |
| pAKH775 | pGP564 (*LEU2; 2μ)* | +/- 500 nucleotides around *TIF1* | This study |
| pAKH776 | pGP564 (*LEU2; 2μ)* | +/- 500 nucleotides around *DED1* | This study |
| pET-22b(+)-*DED1* | pET-22b(+) | wt His_6_-Ded1p CDS | Iost et al., 1999 |
| pET-22b(+)-*DED1*^E307A^ | pET-22b(+) | His_6_-Ded1p^E307A^ CDS | Iost et al., 1999 |
| pET-22b(+)-His-Ded1-HA | pET-22b(+) | His_6_- Ded1-HACDS | This study |
| pET-22b(+)-His-*DED1*^T408I^ | pET-22b(+) | His_6_-Ded1p^T408I^ CDS | This study |
| pET-22b(+)-His-*DED1*^1-535^ | pET-22b(+) | His_6_-Ded1p^1-535^ CDS | Hilliker et al., 2011 |
| pET41a(+)-GST-S•Tag-eIF4G1-His | pET41a(+) | GST-S-Tag-eIF4G1-His_6_ CDS | Hilliker et al., 2011 |
| pET21b(+)-eIF4E | pET-22b(+) | eIF4E CDS | Hilliker et al., 2011 |
| pET-22b(+)-His_6_-thrombin-SUMO- *DED1*^117-604^ | pET-22b(+)-His_6_-thrombin-SUMO | Ded1p^117-604^ CDS | This study |
| pET-28a(+)-eIF4A | pET-28a(+) | eIF4A CDS | Schütz et al., 2008 |
| pET-28a(+)-eIF4A^E171A^ | pET-28a(+) | eIF4A^E171A^  CDS | This study |
| pET-41a(+)-eIF4A | pET-41a(+) | GST-His_6_-eIF4A CDS | This study |
| pEJ4 | pCRII-TOPO | Genomic region of *HIS3-DED1* | This study. |
| pEJ6 | pCRII-TOPO | Genomic region of *HIS3-DED1* with  *ded1-95* allele | This study. |
| pEJ13 | pET-22b(+)-*DED1*^T408I^ | Expression plasmid of N-terminally tagged His_6_-Ded1p with *ded1-95* allele | This study. |
| pEJ10 | pRS413 (*HIS3; CEN*) | *DED1*^117-604^ | This study. |

**Supplementary file 4. Yeast strains**

| **Strain** | **Genotype** | **Reference or source** |
| --- | --- | --- |
| BY4741 | *MATa his3Δ1 leu2Δ0 met15Δ0 ura3Δ0* | GE Healthcare |
| yRP2799 | *MATa his3Δ1, leu2Δ0, lys2Δ0, met15Δ0, ura3Δ0, ded1::KANMX (DED1-URA3)* | Hilliker et al., 2011 |
| yEJ1 | *MATa leu2Δ0 met15Δ0 ura3Δ0* | This study. |
| yEJ3 | *MATa leu2Δ0 met15Δ0 ura3Δ0 ded1-95* | This study. |
