# Supplementary material for: Coupling between the DEAD-box RNA helicases Ded1p and eIF4A
Source: eLife. 2016 Aug 5;5:e16408. doi: 10.7554/eLife.16408 (PMC4990422; doi:10.7554/eLife.16408)
Supplement: Supplementary file 2. — DOI: http://dx.doi.org/10.7554/eLife.16408.023 [file elife-16408-supp2.docx]

**Supplementary file 2**

**Parameters calculated from the thermodynamic model (Figure 4A)**

2A - Unwinding rate constants (k_unw_) for the unwinding-competent complexes.
2B - Equilibrium dissociation constants (K_1/2_) for complexes containing only eIF4A.

2C - Equilibrium dissociation constants for complexes containing only eIF4A-eIF4G.
2D - Equilibrium dissociation constants for complexes containing only Ded1p - eIF4A.
2E - Equilibrium dissociation constants for complexes containing Ded1p - eIF4G.
2F - Equilibrium dissociation constants for complexes containing Ded1p - eIF4G - eIF4A.

**Supplementary file 2A.
Unwinding rate constants (k_unw_) for the unwinding-competent complexes**

| **Complex** | **k_unw_ (min^-1^)** | **k_unw_ range (min^-1^)**  (95% Confidence Interval) |
| --- | --- | --- |
| *16 bp duplex, 3’-25 ntssRNA overhang* | | |
| D3TR | 28 | 24.4 - 31.4 |
| ATR | 0.016 | 0.015 - 0.044 |
| GATR | 0.014 | 0.010 - 0.034 |
| DTGR | 0.18 | 0.046 - 0.3 |
| DTA(T)R | 33 | 21 - 53 |
| DTGATR | 24 | 18 - 31 |
| *10 bp duplex, 3’-25 ntssRNA overhang* | | |
| ATR | 0.091 | 0.082 - 0.26 |
| GATR | 1.2·10^-4^ | (0.012 - 1.7)·10^-4^ |

Unwinding rate constants at enzyme and ATP saturation and associated confidence intervals, calculated with the thermodynamic model (***Figure 4A***). Abbreviations: D3 - Ded1p trimer, D - Ded1p, T - ATP, R - RNA, A - eIF4A, G - eIF4G.

**Supplementary file 2B.
Equilibrium dissociation constants (K_1/2_) for complexes containing only eIF4A.**

|  | **Complex** | **Binding Step** | **K_1/2_ (nM)** | **K_1/2_range (nM)**  (95% Confidence Interval) |
| --- | --- | --- | --- | --- |
| -RNA | AT | A + T | 0.15·10^6^ | (0.12-0.35)·10^6^ |
| +RNA | AR | R + A | 6,000 | >5,000 |
|  | ATR | AT + R | 6,000 | >2,500 |
|  | ATR | AR + T | 0.15·10^6^ | (0.13-0.19)·10^6^ |

Equilibrium dissociation constants at enzyme and ATP saturation and associated confidence intervals, calculated with the thermodynamic model (***Figure 4A***). Abbreviations: T - ATP, R - RNA, A - eIF4A

**Supplementary file 2C.
Equilibrium dissociation constants for complexes containing only eIF4A-eIF4G.**

|  | **Complex** | **Binding Step** | **K_1/2_ (nM)** | **K_1/2_range (nM)**  (95% Confidence Interval) |
| --- | --- | --- | --- | --- |
| -RNA | GA | G + A | 500 | 200-1,300 |
|  | GAT | AT + G | 1,000 | 390-2,600 |
|  | GAT | GA + T | 0.3·10^6^ | (0.23-0.71)·10^6^ |
| +RNA | GR | R + G | 140 | 125-155 |
|  | GAR | G + AR | 25 | 9.7-63 |
|  | GAR | GR + A | 1,060 | 420-2,700 |
|  | GAR | GA + R | 300 | 120-750 |
|  | GATR | G + ATR | 49 | 19-130 |
|  | GATR | GR + AT | 2,100 | 832-5,400 |
|  | GATR | GAT + R | 300 | 120-750 |
|  | GATR | GAR + T | 0.3·10^6^ | (0.23-0.71)·10^6^ |

Equilibrium dissociation constants at enzyme and ATP saturation and associated confidence intervals, calculated with the thermodynamic model (***Figure 4A***). Abbreviations: T - ATP, R - RNA, A - eIF4A, G - eIF4G.

**Supplementary file 2D.
Equilibrium dissociation constants for complexes containing only Ded1p - eIF4A**

|  | **Complex** | **Binding Step** | **K_1/2_ (nM)** | **K_1/2_range (nM)**  (95% Confidence Interval) |
| --- | --- | --- | --- | --- |
| -RNA | DA | D + A | 800 | 440-2,400 |
|  | DTA | DT + A | 800 | 440-2,400 |
|  | DTA | DA + T | 1.6·10^6^ | NV ^(a)^ |
|  | DAT | D + AT | 800 | 340-2,400 |
|  | DAT | DA + T | 150 | 120-350 |
|  | DTAT | DT + AT | 800 | 340-2,400 |
|  | DTAT | DTA + T | 150 | 120-350 |
|  | DTAT | DAT + T | 1.6·10^6^ | NV ^(a)^ |
| +RNA | DAR | D + AR | 45 | 25-135 |
|  | DAR | DR + A | 270 | 150-810 |
|  | DAR | DA + R | 330 | 110-600 |
|  | DTAR | DT + AR | 89 | 50-270 |
|  | DTAR | DTR + A | 710 | 400-2,200 |
|  | DTAR | DTA + R | 670 | 220-1,200 |
|  | DTAR | DAR + T | 3.2·10^6^ | >2.6·10^6^ |
|  | DATR | D + ATR | 45 | 25-135 |
|  | DATR | DR + AT | 270 | 110-810 |
|  | DATR | DAT + R | 330 | 110-600 |
|  | DATR | DAR + T | 150 | 120-350 |
|  | DTATR | DTAT + R | 670 | 220-1,200 |
|  | DTATR | DTR + AT | 710 | 300-2,200 |
|  | DTATR | ATR + DT | 89 | 50-270 |
|  | DTATR | DTAR + T | 150 | 120-350 |
|  | DTATR | DATR + T | 3.2·10^6^ | >2.6·10^6^ |

Equilibrium dissociation constants at enzyme and ATP saturation and associated confidence intervals, calculated with the thermodynamic model (***Figure 4A***). Abbreviations: D - Ded1p, T - ATP, R - RNA, A - eIF4A).

^(a)^ not varied parameter

**Supplementary file 2E.
Equilibrium dissociation constants for complexes containing Ded1p - eIF4G.**

|  | **Complex** | **Binding Step** | **K_1/2_ (nM)** | **K_1/2_range (nM)**  (95% Confidence Interval) |
| --- | --- | --- | --- | --- |
| -RNA | DG | D + G | 2,500 | 1,400-5,400 |
|  | DTG | DT + G | 2,500 | 1,400-5,400 |
|  | DTG | DG + T | 1.6·10^6^ | NV ^(a)^ |
| +RNA | DGR | D + GR | 49 | 27-100 |
|  | DGR | DR +G | 6.8 | 3.8-15 |
|  | DGR | DG + R | 2.7 | 1.3-4.9 |
|  | DTGR | DTR + G | 18 | 10-39 |
|  | DTGR | DT + GR | 98 | 54-210 |
|  | DTGR | DTG + R | 5.5 | 2.6-9.9 |
|  | DTGR | DGR + T | 3.2·10^6^ | >3.1·10^6^ |

Equilibrium dissociation constants at enzyme and ATP saturation and associated confidence intervals, calculated with the thermodynamic model (***Figure 4A***). Abbreviations: D - Ded1p, T - ATP, R - RNA, G - eIF4G).

^(a)^ not varied parameter

**Supplementary file 2F.
Equilibrium dissociation constants for complexes containing Ded1p - eIF4G - eIF4A**

|  | **Complex** | **Binding Step** | **K_1/2_ (nM)** | **K_1/2_range (nM)**  **(95% Confidence Interval)** |
| --- | --- | --- | --- | --- |
| -RNA | DGA | D + GA | 0.69 | 0.28-1.5 |
|  | DGA | DA + G | 0.43 | 0.10- 0.80 |
|  | DGA | DG + A | 0.14 | 0.040- 0.30 |
|  | DTGA | DT + GA | 0.69 | 0.28-1.5 |
|  | DTGA | DTA + G | 0.43 | 0.10- 0.80 |
|  | DTGA | DTG + A | 0.14 | 0.04- 0.30 |
|  | DTGA | DGA + T | 1.6·10^6^ | NV ^(a)^ |
|  | DGAT | D + GAT | 0.69 | 0.28-1.5 |
|  | DGAT | DAT + G | 0.86 | 0.21-1.6 |
|  | DGAT | DG + AT | 0.28 | 0.08- 0.61 |
|  | DGAT | DGA + T | 300 | 230-710 |
|  | DTGAT | DTAT + G | 0.86 | 0.21-1.6 |
|  | DTGAT | GAT+DT | 0.69 | 0.28-1.5 |
|  | DTGAT | DTG+AT | 0.28 | 0.08- 0.61 |
|  | DTGAT | DTGA + T | 300 | 230-710 |
|  | DTGAT | DGAT + T | 3.2·10^6^ | >3.1·10^6^ |
| +RNA | DGAR | D + GAR | 0.33 | 0.18- 0.98 |
|  | DGAR | DR + GA | 0.10 | 0.03- 0.26 |
|  | DGAR | DGR + A | 7.0 | 2.1-16 |
|  | DGAR | DAR + G | 0.18 | 0.05- 0.34 |
|  | DGAR | DG + AR | 0.003 | 0.001- 0.009 |
|  | DGAR | DA + GR | 0.43 | 0.13-1.0 |
|  | DGAR | DGA + R | 140 | 68-440 |
|  | DTGAR | G + DTAR | 0.18 | 0.05- 0.34 |
|  | DTGAR | A + DTGR | 7.0 | 2.1-16 |
|  | DTGAR | R + DTGA | 280 | 140-890 |
|  | DTGAR | DT + GAR | 0.65 | 0.36-2.0 |
|  | DTGAR | GR + DTA | 0.86 | 0.26-2.0 |
|  | DTGAR | GA + DTR | 0.26 | 0.09- 0.71 |
|  | DTGAR | AR + DTG | 0.006 | 0.002- 0.018 |
|  | DTGAR | T + DGAR | 3.2·10^6^ | >1.3·10^6^ |
|  | DGATR | D + GATR | 0.33 | 0.18- 0.98 |
|  | DGATR | G + DATR | 0.36 | 0.10- 0.68 |
|  | DGATR | R + DGAT | 140 | 53-350 |
|  | DGATR | DR + GAT | 0.10 | 0.03- 0.21 |
|  | DGATR | DG + ATR | 0.006 | 0.002- 0.014 |
|  | DGATR | GR + DAT | 0.86 | 0.20-1.6 |
|  | DGATR | AT + DGR | 14 | 3.3-25 |
|  | DGATR | T + DGAR | 0.3·10^6^ | (0.23-0.71)·10^6^ |
|  | DTGATR | G + DTATR | 0.36 | 0.10- 0.68 |
|  | DTGATR | R + DTGAT | 280 | 110-690 |
|  | DTGATR | GR + DTAT | 1.7 | 0.4-3.1 |
|  | DTGATR | DT + GATR | 0.65 | 0.36-2.0 |
|  | DTGATR | AT + DTGR | 14 | 3.3-25 |
|  | DTGATR | DTR + GAT | 0.26 | 0.07- 0.55 |
|  | DTGATR | ATR + DTG | 0.013 | 0.004- 0.028 |
|  | DTGATR | T + DTGAR | 0.3·10^6^ | (0.23-0.71)·10^6^ |
|  | DTGATR | T + DGATR | 3.2·10^6^ | >1.3·10^6^ |

Equilibrium dissociation constants at enzyme and ATP saturation and associated confidence intervals, calculated with the thermodynamic model (***Figure 4A***). (Abbreviations: D - Ded1p, T - ATP, R - RNA, A- eIF4A, G - eIF4G).
