# Supplementary material for: Coupling between the DEAD-box RNA helicases Ded1p and eIF4A
Source: eLife. 2016 Aug 5;5:e16408. doi: 10.7554/eLife.16408 (PMC4990422; doi:10.7554/eLife.16408)
Supplement: Supplementary file 3. — DOI: http://dx.doi.org/10.7554/eLife.16408.024 [file elife-16408-supp3.docx]

**Supplementary file 3**

**Thermodynamically linked binding steps used for modeling.**

| **G-A** | **D-A** | **D-G** | **D-G-A** |
| --- | --- | --- | --- |
| G + A | D + A | D + G | D + GA |
| AT + G | DT + A | DT + G | DA + G |
| G + AR | D + AT | D + GR | DG + A |
| GR + A | DT + AT | DR + G | DT + GA |
| G + ATR | D + AR | DTR + G | DTA + G |
| GR + AT | DR + A | DT + GR | DTG + A |
| DA + G | DT + AR | D + GA | D + GAT |
| DG + A | DTR + A | DA + G | DAT + G |
| DTA + G | D + ATR | DT + GA | DG + AT |
| DTG + A | DR + AT | DTA + G | DTAT + G |
| DAT + G | DTR + AT | D + GAT | GAT+DT |
| DG + AT | ATR + DT | DAT + G | DTG+AT |
| DTAT + G | D + GA | DTAT + G | D + GAR |
| DTG+AT | DG + A | GAT+DT | DR + GA |
| DGR + A | DT + GA | D + GAR | DGR + A |
| DAR + G | DTG + A | DR + GA | DAR + G |
| DG + AR | D + GAT | DAR + G | DG + AR |
| DA + GR | DG + AT | DA + GR | DA + GR |
| G + DTAR | GAT+DT | G + DTAR | G + DTAR |
| A + DTGR | DTG+AT | DT + GAR | A + DTGR |
| GR + DTA | D + GAR | GR + DTA | DT + GAR |
| AR + DTG | DR + GA | GA + DTR | GR + DTA |
| G + DATR | DGR + A | D + GATR | GA + DTR |
| DG + ATR | DG + AR | G + DATR | AR + DTG |
| GR + DAT | A + DTGR | DR + GAT | D + GATR |
| AT + DGR | DT + GAR | GR + DAT | G + DATR |
| G + DTATR | GA + DTR | G + DTATR | DR + GAT |
| GR + DTAT | AR + DTG | GR + DTAT | DG + ATR |
| AT + DTGR | D + GATR | DT + GATR | GR + DAT |
| ATR + DTG | DR + GAT | DTR + GAT | AT + DGR |
|  | DG + ATR |  | G + DTATR |
|  | AT + DGR |  | GR + DTAT |
|  | DT + GATR |  | DT + GATR |
|  | AT + DTGR |  | AT + DTGR |
|  | DTR + GAT |  | DTR + GAT |
|  | ATR + DTG |  | ATR + DTG |

**Supplementary file 3A.
Thermodynamically linked binding steps involving protein complexes.**

Each column represents a set of thermodynamically linked binding steps, used in the modeling. The bold letters at the top of each column represent the components present in each step in the linked set. For example: G-A includes thermodynamically linked binding steps to form complexes which contain eIF4A and eIF4G. Abbreviations: D - Ded1p, T - ATP, R - RNA, A- eIF4A, G - eIF4G.

**Supplementary file 3B.
Thermodynamically linked binding steps involving RNA-protein complexes.**

| **A-R** | **G-A-R** | **D-A-R** | **D-G-R** | **D-G-A-R** |
| --- | --- | --- | --- | --- |
| R + A | G + AR | D + AR | D + GR | D + GAR |
| AT + R | GR + A | DR + A | DR + G | DR + GA |
| GR + A | GA + R | DA + R | DG + R | DGR + A |
| GA + R | G + ATR | DT + AR | DTR + G | DAR + G |
| GR + AT | GR + AT | DTR + A | DT + GR | DG + AR |
| GAT + R | GAT + R | DTA + R | DTG + R | DA + GR |
| DR + A | DR + GA | D + ATR | D + GAR | DGA + R |
| DA + R | DGR + A | DR + AT | DR + GA | G + DTAR |
| DTR + A | DAR + G | DAT + R | DAR + G | A + DTGR |
| DTA + R | DG + AR | DTAT + R | DG + AR | R + DTGA |
| DR + AT | DA + GR | DTR + AT | DA + GR | DT + GAR |
| DAT + R | DGA + R | ATR + DT | DGA + R | GR + DTA |
| DTAT + R | G + DTAR | D + GAR | G + DTAR | GA + DTR |
| DTR + AT | A + DTGR | DR + GA | R + DTGA | AR + DTG |
| DR + GA | R + DTGA | DGR + A | DT + GAR | D + GATR |
| DGR + A | GR + DTA | DG + AR | GR + DTA | G + DATR |
| DA + GR | GA + DTR | DA + GR | GA + DTR | R + DGAT |
| DGA + R | AR + DTG | DGA + R | AR + DTG | DR + GAT |
| A + DTGR | G + DATR | A + DTGR | D + GATR | DG + ATR |
| R + DTGA | R + DGAT | R + DTGA | G + DATR | GR + DAT |
| GR + DTA | DR + GAT | DT + GAR | R + DGAT | AT + DGR |
| GA + DTR | DG + ATR | GR + DTA | DR + GAT | G + DTATR |
| R + DGAT | GR + DAT | GA + DTR | DG + ATR | R + DTGAT |
| DR + GAT | AT + DGR | AR + DTG | GR + DAT | GR + DTAT |
| GR + DAT | G + DTATR | D + GATR | G + DTATR | DT + GATR |
| AT + DGR | R + DTGAT | R + DGAT | R + DTGAT | AT + DTGR |
| R + DTGAT | GR + DTAT | DR + GAT | GR + DTAT | DTR + GAT |
| GR + DTAT | AT + DTGR | DG + ATR | DT + GATR | ATR + DTG |
| AT + DTGR | DTR + GAT | GR + DAT | DTR + GAT |  |
| DTR + GAT | ATR + DTG | AT + DGR | ATR + DTG |  |
|  |  | R + DTGAT |  |  |
|  |  | GR + DTAT |  |  |
|  |  | DT + GATR |  |  |
|  |  | AT + DTGR |  |  |
|  |  | DTR + GAT |  |  |
|  |  | ATR + DTG |  |  |

Each column represents a set of thermodynamically linked binding steps, used in the modeling. The bold letters at the top of each column represent the components present in each step in the linked set. Abbreviations: D - Ded1p, T - ATP, R - RNA, A- eIF4A, G - eIF4G.

**Supplementary file 3C.
Thermodynamically linked binding steps involving eIF4A-ATP.**

| **A-T** | **G-A-T** | **D-A-T-R** | **D-G-A-T-R** |
| --- | --- | --- | --- |
| A + T | AT + G | D + ATR | D + GATR |
| AR + T | GA + T | DR + AT | G + DATR |
| GA + T | G + ATR | DAT + R | R + DGAT |
| GAR + T | GR + AT | DAR + T | DR + GAT |
| DA + T | GAR + T | DTAT + R | DG + ATR |
| DTA + T | DAT + G | DTR + AT | GR + DAT |
| DAR + T | DG + AT | ATR + DT | AT + DGR |
| DTAR + T | DGA + T | DTAR + T | T + DGAR |
| DGA + T | DTAT + G | D + GATR | G + DTATR |
| DTGA + T | DTG+AT | R + DGAT | R + DTGAT |
| T + DGAR | DTGA + T | DR + GAT | GR + DTAT |
| T + DTGAR | G + DATR | DG + ATR | DT + GATR |
|  | DG + ATR | GR + DAT | AT + DTGR |
|  | GR + DAT | AT + DGR | DTR + GAT |
|  | AT + DGR | T + DGAR | ATR + DTG |
|  | T + DGAR | R + DTGAT | T + DTGAR |
|  | G + DTATR | GR + DTAT |  |
|  | GR + DTAT | DT + GATR |  |
|  | AT + DTGR | AT + DTGR |  |
|  | ATR + DTG | DTR + GAT |  |
|  | T + DTGAR | ATR + DTG |  |
|  |  | T + DTGAR |  |

Each column represents a set of thermodynamically linked binding steps, used in the modeling. The bold letters at the top of each column represent the components present in each step in the linked set. Abbreviations: D - Ded1p, T - ATP, R - RNA, A- eIF4A, G - eIF4G.

**Supplementary file 3D.
Thermodynamically linked binding steps involving Ded1p-ATP.**

| **D-T-A-R** | **D-T-G-R** | **D-T-G-A-R** |
| --- | --- | --- |
| DT + AR | DTR + G | G + DTAR |
| DTR + A | DT + GR | A + DTGR |
| DTA + R | DTG + R | R + DTGA |
| DAR + T | DGR + T | DT + GAR |
| DTAT + R | G + DTAR | GR + DTA |
| DTR + AT | R + DTGA | GA + DTR |
| ATR + DT | DT + GAR | AR + DTG |
| DATR + T | GR + DTA | T + DGAR |
| A + DTGR | GA + DTR | G + DTATR |
| R + DTGA | AR + DTG | R + DTGAT |
| DT + GAR | T + DGAR | GR + DTAT |
| GR + DTA | G + DTATR | DT + GATR |
| GA + DTR | R + DTGAT | AT + DTGR |
| AR + DTG | GR + DTAT | DTR + GAT |
| T + DGAR | DT + GATR | ATR + DTG |
| R + DTGAT | DTR + GAT | T + DGATR |
| GR + DTAT | ATR + DTG |  |
| DT + GATR | T + DGATR |  |
| AT + DTGR |  |  |
| DTR + GAT |  |  |
| ATR + DTG |  |  |
| T + DGATR |  |  |

Each column represents a set of thermodynamically linked binding steps, used in the modeling. The bold letters at the top of each column represent the components present in each step in the linked set. Abbreviations: D - Ded1p, T - ATP, R - RNA, A- eIF4A, G - eIF4G.
